# Research on an Efficient Barrier Adjustment Method for Bistable Vibration Energy Harvesters Based on a Rhombus Linkage Mechanism

**DOI:** 10.3390/mi17060681

**Published:** 2026-05-30

**Authors:** Lulu Fu, Zhen Xiao, Tao Yu, Guansong Shan, Guanggui Cheng, Jie Song

**Affiliations:** 1 School of Mechanical Engineering, Jiangsu University, Zhenjiang 212013, China; 2 School of Advanced Technology, Xi’an Jiaotong-Liverpool University, Suzhou 215123, China; guansong.shan@xjtlu.edu.cn

**Keywords:** bistable, potential barrier adjustment, rhombus linkage mechanism, broadband, vibration energy harvesting

## Abstract

Although bistable vibration energy harvesters offer promising broadband characteristics, their efficiency is often hindered by fixed potential barriers that confine the system to small-amplitude intra-well motion. The core innovation of this work is the proposal of a synchronous potential barrier regulation mechanism for multiple subsystems based on a rhombus linkage mechanism. This study introduces a novel multi-subsystem bistable vibration energy harvester (MBEH) integrated with a rhombus linkage mechanism to achieve tunable potential barriers. The mechanism facilitates the coupling of four bistable subsystems, where adjusting the magnet spacing of one subsystem allows for the synchronous regulation of magnetic gaps in others. This architecture ensures a continuous and precise optimization of the potential barrier. Consequently, this mechanism yields remarkable performance advancements, achieving highly efficient coupling among subsystems. Furthermore, potential barrier regulation efficiency is substantially increased, while operating bandwidths of subsystems are complementary and superimposed. Results from numerical investigations indicate that at an excitation acceleration of 0.6 g, MBEH outperforms conventional BEH with a 13.58 Hz increase in summed subsystem bandwidth and a 0.0223 μW gain in output power. The findings validate the efficacy of the proposed MBEH as a high-performance solution for robust broadband vibration energy harvesting.

## 1. Introduction

With the rapid advancement of wireless sensor networks (WSNs) and Internet of Things (IoT) technologies, the application of distributed low-power sensors in industrial monitoring [1,2,3], smart agriculture [4,5,6], and deep space exploration [7] has experienced explosive growth. Such sensors are typically deployed in remote, confined, or extreme environments that are inaccessible to humans. Traditional chemical batteries are constrained by their finite service life and the cumbersome replacement process, while also posing potential environmental pollution risks [8,9]. As a sustainable self-powered strategy, ambient vibration energy harvesting technology enables the conversion of mechanical energy from the environment into stable electrical energy, thereby providing a robust solution to meet the long-term power demands of low-power electronic devices [10,11]. Currently, four mainstream energy conversion mechanisms are employed in vibration energy harvesting: piezoelectric [12], electrostatic [13], electromagnetic [14], and triboelectric effects [15,16]. Among these, piezoelectric energy harvesters (PEHs) have attracted extensive research interest due to their superior characteristics, such as high electromechanical coupling coefficients, high power density, ease of micro-scale fabrication, and excellent integration potential [17,18,19]. However, vibration sources in practical environments generally exhibit characteristics of broadband spectra, weak intensity, and non-stationary randomness. Restricted by their inherent narrow resonant bandwidth, conventional linear PEHs can only maintain high conversion efficiency within a limited frequency range and are highly sensitive to variations in excitation frequencies. These drawbacks significantly limit their adaptability to complex and variable operating conditions. Moreover, under weak vibration environments, their output power and stability are significantly constrained, which severely limits their practical engineering application scope [20,21]. Consequently, overcoming the bandwidth limitations of linear structures to achieve broadband and high-efficiency energy harvesting has become a critical research challenge in this field.

To overcome the bandwidth bottleneck of linear energy harvesters, nonlinear bistable energy harvesters (BEHs) have emerged as a research hotspot in the field of broadband vibration energy harvesting, owing to their unique inter-well dynamic characteristics [22,23,24]. By constructing a potential energy surface with dual potential wells, two stable equilibrium points and a potential barrier between them are established. Large-amplitude inter-well motion can be triggered under external excitation, which significantly broadens the effective bandwidth [25]. Currently, structural bistability is primarily achieved through two technical routes: the mechanical prestress method and the magnetic coupling method. For the former, axial prestress is applied to piezoelectric beams to induce buckling deformation, thereby directly creating a double-well potential configuration [26,27,28,29]. For the latter, attractive or repulsive forces between permanent magnets are utilized to design multi-stable systems and multi-degree-of-freedom coupled structures, effectively expanding the response bandwidth [23,30,31]. In addition, the electromagnetic domain also faces the need for nonlinear bandwidth enhancement; researchers have introduced Duffing nonlinearity via mechanisms like magnetic levitation to improve adaptability to broadband excitations [32,33]. Although these approaches can effectively enhance the broadband adaptability of energy harvesting systems, most existing BEHs adopt fixed structural parameters. The potential barrier height is strictly limited by rigid mechanical constraints, making it difficult to dynamically tune the barrier under weak vibration conditions. Such limitations impede sustained high-energy inter-well motion and render the system unable to cope with the dynamically varying excitation spectra in practical service environments [34,35].

To address this issue, researchers have introduced elastic supports [36] or spring oscillators [37,38,39] into the bistable mechanism to replace rigid mechanical constraints, thereby altering the magnet spacing and reducing the potential barrier height for adaptation to external excitation. Jiang et al. [38] effectively lowered the inter-well transition threshold by adopting composite spring–mass movable joints, which reduced the potential barrier height by 65% and increased the effective bandwidth by 108%. Dang et al. [39] integrated a T-shaped nonlinear beam with a movable magnet to enrich the system’s resonant frequencies and improve the low-frequency response; at an acceleration of 0.3 g and a frequency of 15.7 Hz, the peak output power reached 2.36 mW. Zhou et al. and Fan et al. [40,41] fixed the magnets on a double-ended clamped flexible beam, and continuous variation of the potential energy function was realized through large deformation of the beam. However, despite these successful realizations of potential barrier height adjustment, research has primarily focused on the regulation of a single bistable unit. Consequently, there remains significant scope for enhancing the regulation efficiency and integration level of multi-unit systems.

To further overcome the above limitations, Fu et al. [42] proposed an S-shaped composite piezoelectric beam integrated with multi-magnetic poles and a spring-supported coupling structure. This design achieves synchronous regulation of potential barriers under multimodal conditions, with a maximum effective bandwidth of 6.62 Hz and a peak output power of 2.91 μW. Lian et al. [43] established a magnetic coupling submodule composed of multiple variable-stiffness beams. Through cooperative regulation among subsystems, the output performance of the system under low-frequency and weak vibration conditions is significantly enhanced, and the operating frequency band is expanded by 36.50 times. Furthermore, our research team previously realized dynamic regulation of the instantaneous potential barrier height by designing a coupling relationship between magnet spacing and vibration displacement, which expanded the energy harvesting frequency domain by 373.8%. Meanwhile, electromagnetic energy harvesters are also evolving toward multi-unit high-efficiency integration and optimized transduction mechanisms to boost output performance under complex environments [44,45]. While these multi-unit structures provide innovative insights for high-efficiency and broadband vibration energy harvesting, most current designs still rely on a single regulation mechanism. Consequently, research on cooperative adjustment strategies for potential barrier height among complex subsystems remains insufficient.

Owing to its superior geometric nonlinearity and displacement amplification capabilities, the rhombus linkage mechanism has been widely applied in precision positioning and stiffness regulation [46,47,48]. Inspired by the above research, a multi-subsystem bistable vibration energy harvester (MBEH) with tunable potential barrier height based on a rhombus linkage mechanism is proposed in this study. This design is intended to overcome the low integration degree and difficult cooperative adjustment of potential barrier height in existing multi-subsystem bistable harvesters. The core innovation of this study lies in utilizing the geometric linkage characteristics of the rhombus linkage to achieve global and synchronous potential barrier regulation across multiple bistable subsystems. Specifically, the four bistable subsystems are integrated into a whole through the rhombus linkage mechanism. In contrast to conventional distributed configurations, the presented structure only needs to adjust the permanent magnet spacing in a single subsystem. The rigid linkage transmission of the rhombus linkage mechanism enables synchronous variation of magnet spacing for the other three subsystems, thus achieving global, precise, and synchronous tuning of potential barrier height. Consequently, the proposed MBEH not only significantly reduces the complexity of potential barrier adjustment, but also effectively improves system integration and dynamic adaptability, providing an efficient engineering solution for adapting to complex and time-varying environmental vibrations.

The remainder of this paper is organized as follows. Section 2 details the structural design and operating principle of MBEH, and establishes the relevant theoretical model. In Section 3, parametric numerical simulation analysis is carried out to explore the effects of magnet spacing, beam length, and excitation intensity on potential barrier height and system dynamic response, so as to clarify the optimization direction of overall output performance. In Section 4, an experimental test platform is built. The broadband characteristic of the proposed harvester is verified under fixed frequency excitation, and a comparative analysis between simulation results and experimental measurements is conducted. Section 5 summarizes the major conclusions of this study, and discusses the application prospects and subsequent improvement directions of the presented structure in practical engineering scenarios.

## 2. Structural Design and Theoretical Modeling

### 2.1. Working Principle

**Figure 1:**
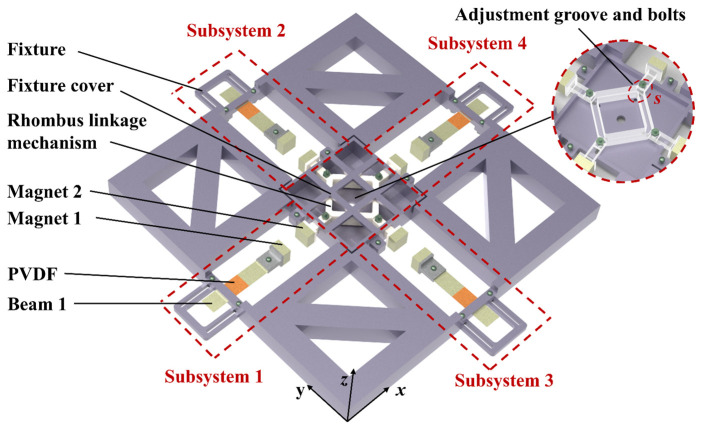
Schematic diagram of the bistable vibration energy harvester with tunable potential barrier height based on a rhombus linkage mechanism (MBEH).

By employing a rhombus linkage mechanism, a multi-subsystem bistable vibration energy harvester (MBEH) with tunable potential barrier height is developed in this work, and its overall configuration is illustrated in Figure 1. The MBEH mainly consists of four magnetic-repulsion bistable subsystems and a rhombus linkage mechanism. All subsystems are evenly distributed around the rhombus linkage mechanism. Each subsystem adopts a cantilever beam structure coupled by permanent magnetic repulsion to construct bistable characteristics and induce nonlinear inter-well dynamic responses. The rhombus linkage mechanism is mounted on a fixture with built-in adjusting grooves. Cooperating with locking bolts, it can achieve precise displacement adjustment. By adjusting the bolt position, the magnet spacing of each subsystem can be synchronously altered, thus accomplishing continuous regulation of the potential barrier height. This design enables the harvester to adapt to environmental vibration excitation with different intensities and frequencies. For comparative analysis, a conventional fixed bistable energy harvester with identical structural parameters is selected as the control model, termed BEH. In contrast, the permanent magnet position of conventional BEH is fixed, and its potential barrier height cannot be actively regulated. The performance advantages of the proposed MBEH in energy harvesting and potential barrier adjustment under weak excitation and broadband vibration environments are quantitatively verified through comparative analysis of the two structures.

### 2.2. Theoretical Model of MBEH

**Figure 2:**
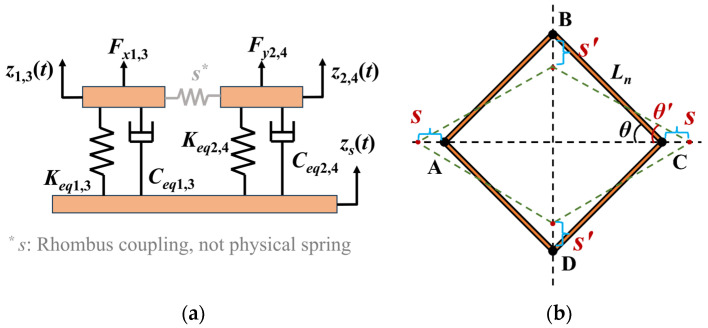
Theoretical model and linkage geometry of MBEH. (**a**) Lumped parameter model of the system. The parameter *s* represents magnet spacing displacement, and the corresponding symbol reflects equivalent coupling stiffness of rhombus linkage instead of actual physical spring. (**b**) Geometric analysis of the rhombus linkage mechanism.

To facilitate the subsequent analysis, the four subsystems are categorized into two groups (subsystems 1, 3 and 2, 4). Under the base excitation *z_s_*(*t*), the first-order bending mode of each cantilever beam subsystem dominates. Consequently, each subsystem is simplified into an equivalent mass–spring–damper system, with the root of the cantilever beam considered as a clamped boundary condition. The lumped parameter model of the system is illustrated in Figure 2a. Based on this model, the equations of motion for the subsystems can be derived as follows:

(1) $$M_{eqi}{\ddot{z}}_{i}(t)+C_{eqi}{\dot{z}}_{i}(t)+K_{eqi}z_{i}(t)-\vartheta_{i}V_{i}(t)=F_{zi}-M_{eqi}{\ddot{z}}_{b}(t)\;(i=1,2,3,4)$$

where the subscript *i* denotes subsystems 1, 2, 3, and 4; for each subsystem *i*, *z_i_*(*t*) represents the vibration amplitude at the free end of the cantilever beam, and *F_zi_* is the magnetic force acting along the *z*-direction. *M_eqi_*, *C_eqi_*, and *K_eqi_* represent the equivalent mass, equivalent damping, and equivalent stiffness, respectively. Specifically, the equivalent mass is expressed as $M_{\mathrm{eqi}}=\frac{33}{140}\left(M_{P}+M_{\mathrm{Ci}}\right)+M_{m}$, where *M_P_* is the mass of the piezoelectric material, *M_Ci_* is the mass of cantilever beam *i*, and *M_m_* is the mass of the magnet. Meanwhile, the equivalent stiffness is defined as $K_{\mathrm{eqi}}=\frac{E_{C}WH^{3}}{4L^{3}}$, where *E_C_* is the elastic modulus of the cantilever beam material, while *W* and *H* denote the width and thickness of the cantilever beam, respectively. Additionally, *ϑ_i_* denotes the electromechanical coupling coefficient of the piezoelectric material, which is defined as $\vartheta_{i}=\frac{d_{31}}{s_{11}^{E}}W(H_{P}+H)$, where *d*_31_ is the piezoelectric constant, $s_{11}^{E}$ is the elastic compliance coefficient, *H_P_* is the thickness of the piezoelectric material, and *H* is the thickness of the cantilever beam.

Furthermore, the equivalent damping *C_eqi_* is defined by mechanical vibration theory as $C_{\mathrm{eqi}}=2\zeta_{i}\sqrt{M_{\mathrm{eqi}}K_{\mathrm{eqi}}}$, where *ζ_i_* represents the equivalent damping ratio. Referring to the damping ratio range of 0.08–0.12 reported in variable potential barrier bistable research [49], and considering the damping enhancement effect caused by bistable motion [50], the damping ratio in this study is set to *ζ_i_* = 0.11. By substituting *M_eqi_* = 0.00343 kg and *K_eqi_* = 60.625 N/m, the equivalent damping is determined as *C_eqi_* = 0.1 N·s/m. This value serves as a theoretical estimate. Furthermore, the same damping assumption is applied to both MBEH and conventional BEH.

The piezoelectric circuit equation for the subsystems is expressed as follows:

(2) $$C_{pi}{\dot{V}}_{i}(t)+{\left(R_{i}\right)}^{-1}V_{i}(t)+\vartheta_{i}{\dot{z}}_{i}(t)=0\;(i=1,2,3,4)$$

where for each subsystem *i*, *C_pi_* is the intrinsic capacitance of the piezoelectric material, *R_i_* is the equivalent resistance, and *V_i_*(*t*) is the voltage. Simultaneously combining Equations (1) and (2) yields the complete electromechanical coupling model for the total system.

By simplifying each magnet pair as a magnetic dipole, the magnetic force components in the *z*-direction (*F_zi_*) and the *x*- and *y*-directions (*F_xj_*, *F_yk_*) are expressed as follows:

(3) $$F_{zi}=\frac{3\mu_{0}{\vec{m}}_{A}{\vec{m}}_{B}z_{i}(t)}{4\pi {\left(\sqrt{d_{i}^{2}+z_{i}^{2}(t)}\right)}^{5}L_{i}}\left[d_{i}-\sqrt{L_{i}^{2}-z_{i}^{2}(t)}\right.-\frac{d_{i}}{{\left(\sqrt{d_{i}^{2}+z_{i}^{2}(t)}\right)}^{2}}\left.\left(z_{i}^{2}(t)-d_{i}\sqrt{L_{i}^{2}-z_{i}^{2}(t)}\right)\right]\;(i=1,2,3,4)$$

(4) $$\begin{array}{l} F_{xj}=\frac{3\mu_{0}{\vec{m}}_{A}{\vec{m}}_{B}}{4\pi {\left(\sqrt{d_{j}^{2}+z_{j}^{2}(t)}\right)}^{5}L_{j}}\left[z_{j}^{2}(t)-3d_{j}\sqrt{L_{j}^{2}-z_{j}^{2}(t)}\right.-\frac{5d_{j}}{{\left(\sqrt{d_{j}^{2}+z_{j}^{2}(t)}\right)}^{2}}\left.\left(z_{j}^{2}(t)-d_{j}\sqrt{L_{j}^{2}-z_{j}^{2}(t)}\right)\right]\\ F_{yk}=\frac{3\mu_{0}{\vec{m}}_{A}{\vec{m}}_{B}}{4\pi {\left(\sqrt{d_{k}^{2}+z_{k}^{2}(t)}\right)}^{5}L_{k}}\left[z_{k}^{2}(t)-3d_{k}\sqrt{L_{k}^{2}-z_{k}^{2}(t)}\right.\left.-\frac{5d_{k}}{{\left(\sqrt{d_{k}^{2}+z_{k}^{2}(t)}\right)}^{2}}\left(z_{k}^{2}(t)-d_{k}\sqrt{L_{k}^{2}-z_{k}^{2}(t)}\right)\right] \end{array}\;(j=1,3,k=2,4)$$

where *μ*_0_ is the permeability of vacuum, and ${\vec{m}}_{A},{\vec{m}}_{B}$ are the magnetic moments of the two magnetic dipoles, which are defined as ${\vec{m}}_{A}=M_{A}\cdot V_{A}\cdot {\hat{m}}_{A},{\vec{m}}_{B}=M_{B}\cdot V_{B}\cdot {\hat{m}}_{B}$. Here, *M_A_* and *M_B_* represent the magnetization intensities, *V_A_* and *V_B_* denote the volumes of the two magnetic dipoles, and ${\hat{m}}_{A},{\hat{m}}_{B}$ are the unit vectors along the directions of the two magnetic dipoles ${\vec{m}}_{A},{\vec{m}}_{B}$, respectively. Additionally, *d_i_* is the magnet spacing for each subsystem *i*, and *L_i_* is the length of the cantilever beam *i*.

It should be noted that calculating the magnetic force between permanent magnets of finite size using the magnetic dipole model is an approximation. Studies show that when the magnet spacing is within the same order of magnitude as the magnet size, the dipole model introduces certain errors [51]. In this study, the ratio of the magnet spacing *d_i_* (12–17 mm) to the magnet size (about 9 mm) is approximately 1.3–1.9, which falls within the near-field range. Therefore, this model introduces some computational deviations. To improve simulation accuracy within this range, a near-field correction coefficient can be introduced [52]. However, the primary objective of this study is to reveal the synergistic regulation mechanism of the rhombus linkage mechanism on the potential barrier height. This work focuses on the variation trend of magnetic force with respect to magnet spacing and displacement, rather than the absolute force values. The dipole model correctly reflects this trend with high computational efficiency, facilitating parametric analysis. Consequently, it is widely adopted in most studies within the multi-stable vibration energy harvesting field [53]. Future work can utilize the equivalent magnetic charge model or finite element method to further improve the calculation accuracy of the magnetic force.

When the magnet spacing of one pair of subsystems (taking subsystems 1 and 3 as an example) is simultaneously adjusted, the adjustment amount of magnet spacing is defined as *s*. In this study, the connecting rods of the rhombus linkage mechanism are considered ideal rigid components that only transmit geometric constraints, and their self-deformation is neglected. Due to the force transmission of the connecting rods in the rhombus linkage mechanism, the magnet spacing of the other pair of subsystems (subsystems 2 and 4) changes synchronously, and the corresponding adjustment is denoted as *s*′. This geometric relationship is illustrated in Figure 2b. The included angle between the two adjacent connecting rods of the rhombus linkage mechanism is initially 90°. Define *θ* as the half-angle between the two connecting rods of the initial rhombus linkage mechanism, and *θ*′ as the half-angle after the adjustment of magnet spacing. Based on the geometric and kinematic constraints, the relationship between *s*′ and *θ*′, as well as the relationship between *θ*′ and *s*, can be established:

(5) $$s^{\prime }=\sin45^{\circ }L_{n}-\sin\theta^{\prime }L_{n}$$

(6) $$\theta^{\prime }=\arccos(\frac{\frac{\sqrt{2}}{2}L_{n}-s}{L_{n}})=\arccos(\frac{\sqrt{2}L_{n}-2s}{2L_{n}})$$

By combining these equations, the relationship between *s*′ and *s* can be obtained:

(7) $$s^{\prime }=\sin45^{\circ }L_{n}-\sin\theta^{\prime }L_{n}=\frac{\sqrt{2}}{2}L_{n}-L_{n}\sin(\arccos(\frac{\sqrt{2}L_{n}-2s}{2L_{n}}))$$

Therefore, the variation relationship of the magnet spacing between subsystems 1 and 3 and subsystems 2 and 4 is expressed as Equation (8):

(8) $$\begin{array}{l} d_{1,3}^{\prime }=d_{1,3}-s\\ d_{2,4}^{\prime }=d_{2,4}+s^{\prime }=d_{2,4}+(\frac{\sqrt{2}}{2}L_{n}-L_{n}\sin(\arccos(\frac{\sqrt{2}L_{n}-2s}{2L_{n}}))) \end{array}$$

where *d*_1,3_ and *d*_2,4_ are the initial magnet spacings of subsystems 1 and 3 as well as subsystems 2 and 4, respectively; *d*′_1,3_ and *d*′_2,4_ are the corresponding magnet spacings after adjustment; *L_n_* is the length of the rhombus linkage mechanism rod; and the magnet spacing of the four subsystems in the fixed comparison group structure (abbreviated as BEH) remains unchanged.

The potential energy function of the subsystems is derived as Equation (9), which primarily consists of the elastic potential energy *U_Bi_* and the magnetic potential energy *U_mi_*.

(9) $$U_{i}=U_{Bi}+U_{mi}\;(i=1,2,3,4)$$

The elastic and magnetic potential energies are given by Equations (10) and (11), respectively.

(10) $$U_{Bi}=\int K_{eqi}z_{i}(t)dz$$

(11) $$U_{mi}=\frac{\mu_{0}}{4\pi }\nabla (\frac{{\vec{m}}_{B}{\vec{r}}_{BA}}{{\left(\sqrt{d_{i}^{2}+z_{i}^{2}(t)}\right)}^{2}}){\vec{m}}_{A}=\frac{\mu_{0}m_{A}m_{B}}{4\pi L_{i}{\left(\sqrt{d_{i}^{2}+z_{i}^{2}(t)}\right)}^{3}}\left[-\sqrt{L_{i}^{2}-z_{i}^{2}(t)}-\frac{3d_{i}(z_{i}^{2}(t)-d_{i}\sqrt{L_{i}^{2}-z_{i}^{2}(t)})}{{\left(\sqrt{d_{i}^{2}+z_{i}^{2}(t)}\right)}^{2}}\right]$$

where ∇ is the vector gradient, and ${\vec{r}}_{BA}$ is the position vector from magnetic dipole *B* to *A*.

It can be inferred from the above equations that tuning the magnet spacing *d_i_* in the potential energy function effectively modulates the potential barrier height. By adjusting the magnet spacing of a single subsystem, the potential barrier heights of remaining subsystems are simultaneously tuned. Consequently, the overall adjustment efficiency of the system is significantly enhanced.

## 3. Numerical Simulation and Analysis

The key structural parameters of MBEH are listed in Table 1. To determine the natural frequencies and mode shapes, an eigenfrequency study within the Solid Mechanics module of COMSOL Multiphysics is utilized to analyze the cantilever beam with its tip magnet in each subsystem. A fixed constraint (*u_x_* = *u_y_* = *u_z_* = 0) is applied at the root of the cantilever beam, while the tip magnet is perfectly bonded to the beam via a continuity boundary condition. For meshing, a swept mesh is applied to the beam with three elements through the thickness to ensure the calculation accuracy of the bending stiffness, and an element size of approximately 1 mm along the length and width directions. Local refinement is implemented at the fixed root and the beam–magnet connection zone. The tip magnet is discretized using free tetrahedral meshes with an element size of 1 mm, and all structural elements are configured as quadratic Lagrange elements. The ARPACK eigenvalue solver is employed to extract the first six structural modes. A mesh convergence verification is performed by gradually increasing the mesh density of the beam (thickness, length, and width directions, as well as the locally refined zones) while keeping the magnet mesh size fixed. As the total degrees of freedom increase, the variation in the first-order natural frequency remains below 0.5%, demonstrating the convergence and reliability of the numerical model. The simulation results indicate that the fundamental natural frequency is approximately 17 Hz.

Based on the structural parameters listed in Table 1, the simulation model of MBEH is constructed in the MATLAB Simulink environment using the electromechanical coupling equations based on the lumped parameter model, specifically Equations (1) and (2). The mechanical governing equations and circuit equations for each subsystem are implemented via signal, integrator, and function modules. The four subsystems are coupled through the rhombus linkage mechanism, with their inter-magnet distances satisfying the geometric constraints defined in Equation (8). The Simulink solver is configured as a variable-step ode45 algorithm. Based on step-size convergence tests, the maximum step size is set to 0.001 s, with the relative and absolute error tolerances specified as 1 × 10^−6^ and 1 × 10^−8^, respectively. By adjusting the harmonic excitation parameters, including amplitude and frequency, from the input signal generator, the output voltages of the four subsystems are evaluated. Concurrently, a baseline BEH simulation model is established as a control group, where the inter-magnet distances of the four subsystems remain fixed while all other configurations are identical to those of MBEH. For the frequency sweep analysis, the excitation frequency increments by 1 Hz, and each frequency point is simulated for 20 s. The steady-state response from the final 5 s is extracted to calculate the root-mean-square (RMS) voltage, which subsequently defines the −6 dB operating bandwidth.

To analyze the nonlinear stability and bifurcation behavior of the system, the displacement and velocity signals at the cantilever tip are extracted once the steady state is reached at each frequency sweep point. Phase trajectories are then plotted to observe whether inter-well motion occurs. Concurrently, the state variables are sampled at intervals of the excitation period to construct a Poincaré section, which serves to determine the periodicity of the dynamic responses, such as period-1, subharmonic, or chaotic motions. Combined with the time-domain waveforms of the output voltage, the amplitude fluctuations and periodic characteristics are utilized as auxiliary indicators to comprehensively identify the critical bifurcation frequencies. These critical points mark the dynamic transition from localized intra-well oscillations to large-amplitude inter-well or chaotic motions.

**Figure 3:**
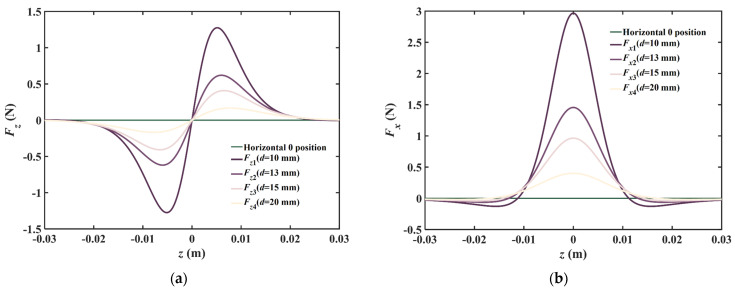
Magnetic force components on the cantilever beam as a function of magnet spacing under vibration. (**a**) Magnetic force in the *z*-direction (*F_z_*). (**b**) Magnetic forces in the *x*- and *y*-directions (*F_x_*, *F_y_*).

Figure 3a,b illustrate the variations in magnetic forces in the *z*-direction (*F_z_*) and the *x*- and *y*-directions (*F_x_*, *F_y_*) with magnet spacing, respectively. It can be observed that the magnetic interaction force between the two magnets changes with the magnet spacing, with an obvious variation in its peak value. This spacing-dependent magnetic force directly alters the nonlinear restoring force characteristics of the system. Consequently, adjusting the magnet spacing can effectively regulate the potential barrier height of the system.

**Figure 4:**
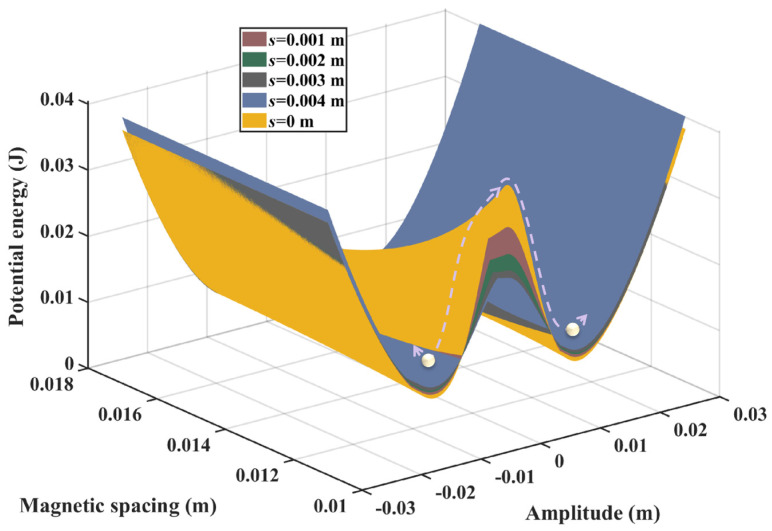
Three-dimensional distribution surface of the subsystem potential energy curve as a function of magnet spacing under different adjustable distances *s*.

Figure 4 presents the three-dimensional distribution surface of the subsystem potential energy curve as a function of magnet spacing under different adjustable distances *s*. When the adjustable distance is 0 mm, the potential barrier height remains extremely high. In this case, the oscillator can hardly cross the potential barrier, and the vibration amplitude is significantly suppressed. After introducing the adjustable distance *s* into the system, the potential barrier height can be effectively reduced. The oscillator can easily achieve inter-well transition, thereby exciting the large-amplitude bistable vibration response. Meanwhile, as the adjustable distance *s* increases, the adjustable range of the potential barrier height is further expanded. The proposed MBEH consists of four vibration subsystems. Benefiting from the linkage characteristic of the rhombus linkage mechanism, adjusting the magnet spacing of a single subsystem can synchronously drive the coordinated variation of the magnet spacing of the remaining subsystems. This design significantly enhances adjustment efficiency of the potential barrier and further improves the overall output performance of the energy harvesting system.

**Figure 5:**
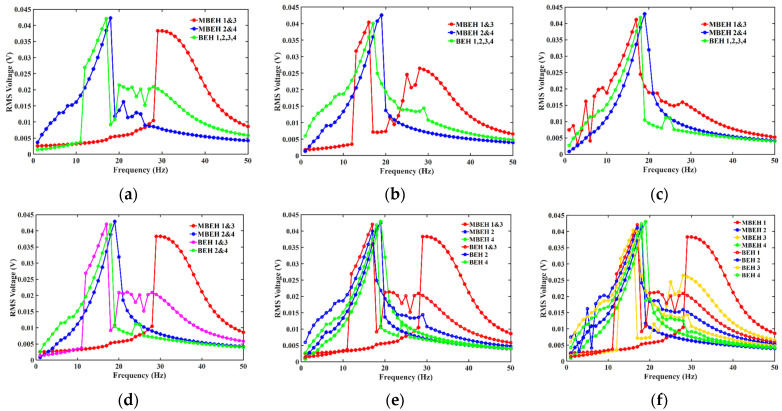
Voltage spectra of MBEH and BEH at different magnet spacings. (**a**) *d*_1,2,3,4_ = 13 mm. (**b**) *d*_1,2,3,4_ = 15 mm. (**c**) *d*_1,2,3,4_ = 17 mm. (**d**) Case D1 with *d*_1,3_ = 13 mm and *d*_2,4_ = 17 mm. (**e**) Case D2 with *d*_1,3_ = 13 mm, *d*_2_ = 15 mm and *d*_4_ = 17 mm. (**f**) Case D3 with *d*_1_ = 13 mm, *d*_2_ = 14 mm, *d*_3_ = 15 mm and *d*_4_ = 16 mm.

Under the condition that the excitation acceleration ${\ddot{z}}_{s}\left(t\right)=0.6\;g$, the adjustable distance *s* = 3 mm, and other parameters of the subsystem are defined in accordance with Table 1, Figure 5 presents the voltage spectra of MBEH and BEH corresponding to different initial magnet spacings. Even when the structural parameters of the cantilever beam in each subsystem remain identical, the resonant frequency of the entire system shifts with the variation of magnet spacing, and the trend of this frequency shift is consistent with that of the magnet spacing change. Specifically, as the magnet spacing decreases, the nonlinear effect of the bistable system is significantly strengthened, and the structure exhibits obvious stiffness softening characteristics, which further leads to a remarkable expansion of the vibration response bandwidth. In contrast, when *d* = 17 mm, the large magnet spacing weakens the bistable effect, resulting in a reduction in the bandwidth broadening capability. Nevertheless, even when the system is not operating at the optimal magnet spacing, the magnet spacing adjustment mechanism of MBEH can still ensure that some of the four subsystems are close to the optimal output state, thereby effectively improving the overall output performance of the energy harvesting system.

**Figure 6:**
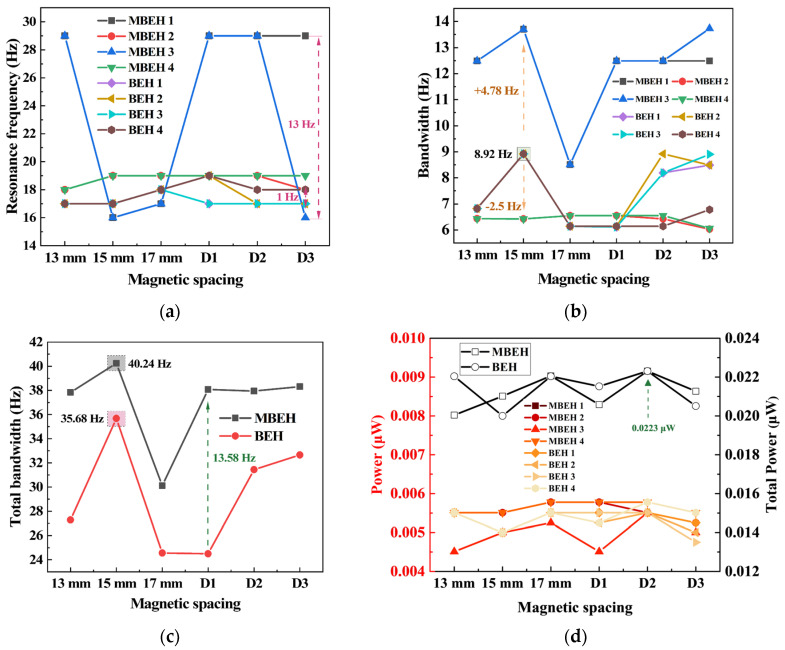
Variation of performance parameters for MBEH and BEH subsystems at different magnet spacings. (**a**) Resonant frequency. (**b**) Subsystem bandwidth. (**c**) Total bandwidth. (**d**) Subsystem power and total power.

Based on Figure 5, the shifts in the resonant frequency of MBEH and BEH under different magnet spacings are analyzed. The corresponding resonant frequency characteristics are presented in Figure 6a, which further verify the analytical conclusions derived from Figure 5. Benefiting from the potential barrier regulation performance of the rhombus linkage mechanism, MBEH delivers a significantly broader frequency tuning range than conventional BEH under most magnet spacing conditions, except for *d* = 17 mm. At this spacing, the large magnet spacing greatly weakens the bistable nonlinear effect and limits the performance improvement.

Among all working conditions, the D3 magnet spacing enables the maximum resonant frequency tuning range of MBEH, reaching 16–29 Hz, while the tuning range of BEH is only 17–18 Hz. The former is an order of magnitude higher than that of the latter, which can effectively cover a wider excitation frequency range. This tuning range represents the system-level bandwidth, which refers to the continuous frequency range that the entire harvester can respond to. Figure 6b further compares the −6 dB operating bandwidth of MBEH and BEH subsystems at various magnet spacings. This bandwidth is defined as the continuous frequency interval where the RMS voltage is not less than 0.5 times its maximum value. Taking *d* = 15 mm as a typical example, it can be seen that although the bandwidth of MBEH subsystems 2 and 4 decreases by 2.5 Hz compared with BEH subsystems 2 and 4, the bandwidth of MBEH subsystems 1 and 3 increases by 4.78 Hz. The overall performance gain outweighs the local bandwidth loss, so MBEH achieves a superior comprehensive bandwidth capacity in contrast to BEH, as illustrated in Figure 6c. Under the D1 magnet spacing condition, the total bandwidth of MBEH, defined as the sum of the bandwidth of its four subsystems, is 13.58 Hz higher than that of BEH, demonstrating outstanding broadband vibration energy harvesting performance. This indicator reflects the capacity for subsystem-level parallel harvesting. Even if the frequency bands of different subsystems overlap, multiple subsystems can simultaneously capture energy within the overlapping frequency band, and each subsystem outputs power independently. Therefore, a larger sum of bandwidth indicates that more energy can be harvested within the same frequency band.

Moreover, under different magnet spacing conditions, the total power of MBEH, calculated as the sum of the power from its four subsystems, is generally superior to that of BEH. Only under a few specific working conditions, due to the influences of resonant coupling and variations in vibrational amplitudes among different groups, BEH exhibits slightly higher power in localized subsystems. This discrepancy stems from the synchronized vibration states across all subsystems in BEH, which ensures relatively high stability in subsystem-level voltage output. In contrast, MBEH adopts a grouped magnetic coupling layout, leading to phase differences and vibrational interference among its subsystems, which causes output fluctuations in localized subsystems. Overall, the total power of MBEH is higher than that of BEH under most working conditions, as summarized in Figure 6d, with a maximum power increase of 0.0223 μW. Consequently, it can be seen that with the adjustable distance fixed at s = 3 mm, the structural advantages of MBEH become increasingly prominent under larger magnet spacings where the bistable effect weakens.

**Figure 7:**
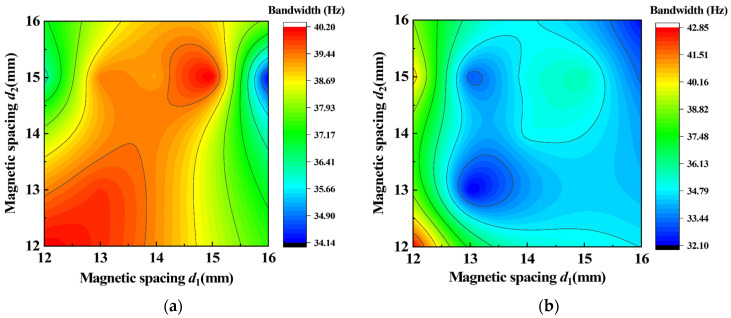

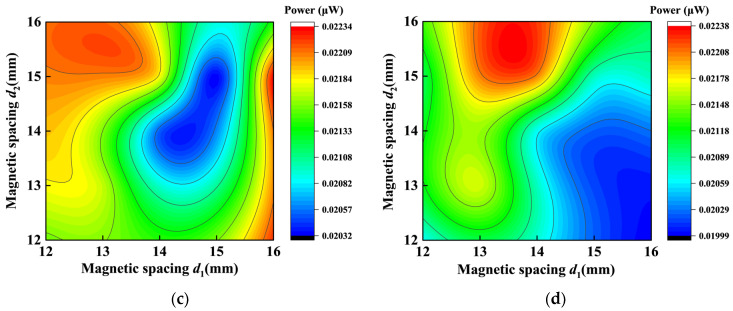
Cloud maps of total bandwidth and total power for MBEH and BEH as a function of subsystem magnet spacings (*d*_1_, *d*_2_). (**a**) Total bandwidth of MBEH. (**b**) Total bandwidth of BEH. (**c**) Total power of MBEH. (**d**) Total power of BEH.

In order to further explore the coupling relationship between the subsystem magnet spacings of MBEH, the variations in total bandwidth and total power of MBEH and BEH are analyzed with subsystem magnet spacing, as shown in Figure 7. Combined with the deformation characteristics of the rhombus linkage mechanism, the subsystems of MBEH are divided into two groups, namely MBEH 1, 3 and MBEH 2, 4. Therefore, only *d*_1_ and *d*_2_ are discussed in this section, while the remaining magnet spacings are fixed at 15 mm.

A comparison between Figure 7a,b indicates that the magnet spacing range for realizing the broadband response of MBEH is far wider than that of BEH under the same working conditions. This comparison also proves that *d*_1_ and *d*_2_ do not independently affect the structural performance, and an obvious coupling effect exists between these two parameters. Due to the coupling effect of the rhombus linkage mechanism, MBEH can achieve excellent broadband performance under a wider variety of parameter combinations. In contrast, BEH requires stricter parameter matching to achieve similar harvesting performance.

The power cloud maps in Figure 7c,d further confirm the structural advantages of MBEH, exhibiting a highly complementary distribution character to the bandwidth cloud maps. The spacing interval corresponding to high power output for MBEH is broader than that for BEH. The high-power region of MBEH is concentrated in the top-left area where *d*_1_ ranges from 12 to 13.5 mm and *d*_2_ ranges from 15 to 16 mm, reaching a peak power of 0.02234 μW, whereas the bandwidth performance in this specific interval is relatively weak. Conversely, within the parameter regions showing superior bandwidth performance, the power output level decreases. For BEH, the power distribution gradient changes uniformly with a small discrepancy between the high and low power intervals, resulting in a limited regulatory scope for output performance via parameter adjustment.

Combined with the previous analysis in Figure 6b, the rhombus linkage mechanism mutually constrains the output performances of different MBEH subsystems. This coupling interaction leads to simultaneous performance enhancements and degradations among these subsystems. Consequently, when optimizing magnet spacing parameters, it is impossible to simultaneously maximize both bandwidth and output power. Instead, adaptive parameters must be selected based on actual working conditions. In contrast, no linkage or coupling interaction exists among BEH subsystems. Their bandwidth and power exhibit unified variation trends, which results in a relatively narrow adjustable performance interval. Crucially, comprehensive comparison reveals that relying on the rhombus linkage coupling structure, MBEH possesses a wider parameter adjustment range and a larger performance regulation space, thereby offering superior adaptability for practical energy harvesting applications.

**Figure 8:**
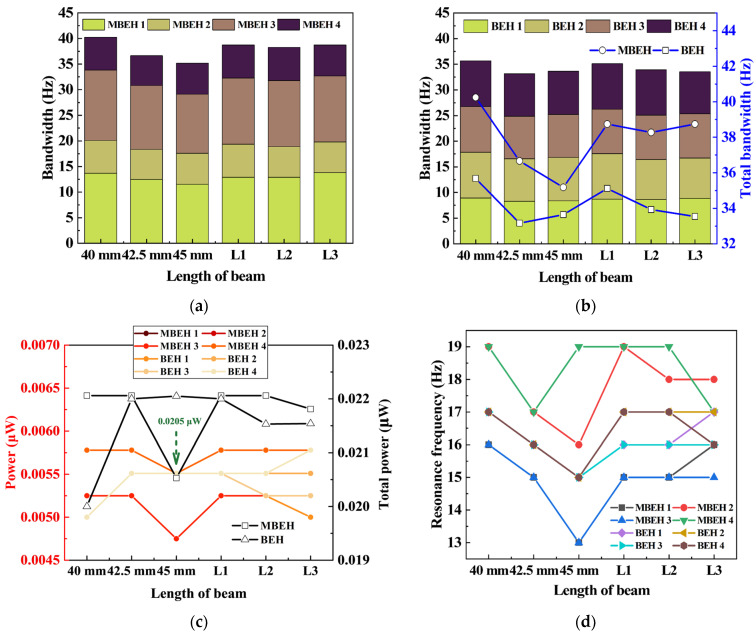
Comparative results of MBEH and BEH under different beam lengths. (**a**) Subsystem bandwidth of MBEH. (**b**) Subsystem bandwidth of BEH and total bandwidth of MBEH and BEH. (**c**) Subsystem power and total power of MBEH and BEH. (**d**) Resonant frequency of MBEH and BEH.

When the excitation acceleration is 0.6 g, the magnet spacing is 15 mm, and the adjustable magnet spacing *s* = 3 mm, the beam length of the subsystem is changed to discuss the influence of the cantilever beam length on the system output. Figure 8 presents the variation laws of the subsystem and total bandwidth, subsystem and total output power, and subsystem frequency shifts of MBEH and BEH under different beam lengths.

It can be seen from Figure 8a,b that compared with BEH, the subsystem bandwidth of each subsystem of MBEH increases and decreases alternately, but the total bandwidth is more advantageous. When the beam length of each subsystem is 40 mm, the total bandwidth of MBEH is 17.84 Hz higher than that of BEH. In addition, it can be observed that under the condition of equal beam length of each subsystem, the smaller the beam length, the more conducive it is to broadening the operating frequency band of the system. However, under the condition of non-uniform combined beam length (each subsystem adopts inconsistent beam length configurations, where the label L1 in the figure denotes *L*_1,3_ = 42 mm and *L*_2,4_ = 40 mm, the label L2 denotes *L*_1,3_ = 42 mm, *L*_2_ = 41 mm and *L*_4_ = 40 mm, and the label L3 denotes *L*_1_ = 40 mm, *L*_2_ = 41 mm, *L*_3_ = 42 mm and *L*_4_ = 43 mm), the operating bandwidth performance of MBEH is more stable than that of BEH.

The variation law of output power with beam length is basically consistent with the trend of bandwidth variation. Only when the beam length is 45 mm, the total output power of MBEH decreases slightly to 0.0205 μW compared with BEH, as shown in Figure 8c, where both the bandwidth and power output of MBEH exhibit a declining trend. Combined with the analysis of the frequency shift law in Figure 8d, it can be found that the resonant frequencies of MBEH 1 and 3 decrease to 13 Hz. The excessively low resonant frequency weakens the energy capture capacity of the structure, which ultimately leads to the decline of the overall output performance of MBEH. Under other beam length conditions, the output power of MBEH is consistently higher than that of BEH, and the power fluctuation amplitude under the non-uniform combined beam length configuration is far smaller than that of BEH. This result indicates that the coordinated linkage of the rhombus linkage mechanism not only broadens the effective operating bandwidth, but also enhances the energy conversion efficiency and performance stability under most working conditions.

**Figure 9:**
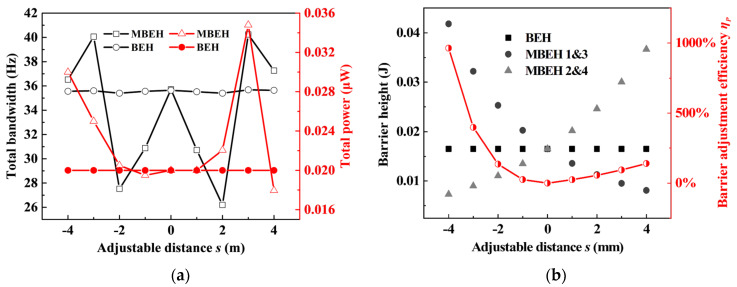
Variation in system performance with adjustable distance *s*. (**a**) Total bandwidth and total power of MBEH and BEH. (**b**) Barrier height and barrier adjustment efficiency of MBEH.

Figure 9a presents the variations in total bandwidth and total power of MBEH and BEH at different adjustable distances *s*. The analysis is conducted under fixed conditions: excitation acceleration ${\ddot{z}}_{s}\left(t\right)=0.6\;g$, uniform beam length *L*_1,2,3,4_ = 40 mm, uniform magnet spacing *d*_1,2,3,4_ = 15 mm, and the excitation frequency set to the resonant frequency of each subsystem. With identical structural parameters, each subsystem of MBEH exhibits bilateral symmetric distribution characteristics. Thus, only MBEH 1 and MBEH 2 are selected as typical representatives to investigate the variation trend of potential barrier height. As illustrated in the figure, when *s* varies within the range of −4 mm to 4 mm, the total bandwidth and total output power of MBEH show a symmetrical distribution centering on *s* = 0 mm. This phenomenon indicates that MBEH can maintain better output performance than BEH regardless of the increase or decrease in adjustable distance. This advantage benefits from the unique deformation characteristics of the rhombus linkage mechanism adopted by MBEH, in which adjacent vertices displace in opposite directions and diagonal vertices displace in the same direction. Such structural features realize the cooperative regulation of potential barriers among multiple subsystems, eliminating the need for separate and independent operations for each subsystem. If the potential barrier adjustment efficiency is defined as $\eta_{P}$, it can be calculated by Equation (12):

(12) $$\eta_{P}=\frac{\sum_{i=1}^{4}\left|\Delta U_{Mi}\right|}{\sum_{i=1}^{2}\left|\Delta U_{Bi}\right|}\times 100\%$$

where Δ*U_Mi_* is the potential barrier height difference of the four MBEH subsystems before and after the adjustment of *s*, and Δ*U_Bi_* is the potential barrier height difference of the two BEH subsystems when the magnet spacing of each is independently offset by *s* (in the increasing direction). This definition is based on the same number of adjustment operations. Here, due to the linkage characteristic of the rhombus linkage mechanism in MBEH, adjusting the magnet spacing of only two subsystems can simultaneously change the spacing of the remaining subsystems. Therefore, two adjustment actions can change the potential barrier heights of all four subsystems. In contrast, each adjustment of conventional BEH can only change the magnet spacing of a single subsystem, meaning that two adjustments can only alter two subsystems. Consequently, the numerator adopts the sum of potential barrier variations of the four MBEH subsystems, while the denominator adopts the sum of potential barrier variations of the two BEH subsystems. The ratio *η_p_* reflects the adjustment effect gain brought by the rhombus linkage mechanism under the same number of adjustment operations.

Figure 9b presents the variation curves of barrier height and barrier adjustment efficiency of MBEH as *s* varies from −4 mm to 4 mm. It can be observed that the potential barrier height is also symmetrically distributed about *s* = 0 mm. Benefiting from the unique deformation and linkage characteristics of the rhombus structure, the potential barrier adjustment efficiency of MBEH is consistently higher than that of BEH, which relies on the independent adjustment of magnet spacing. This result means that under the same adjustable distance *s*, MBEH can generate a larger total potential barrier variation. Consequently, it achieves a more significant enhancement in output performance with fewer adjustment operations, reflecting the practical advantage of the rhombus linkage mechanism in tuning convenience. When *s* < −4 mm, the potential barrier adjustment efficiency presents a continuous increasing trend, whereas the growth rate of the efficiency gradually slows down when *s* > 4 mm. For *s* < −4 mm, the magnet spacing is excessively compressed, leading to a high initial potential barrier height of the system. In contrast, a larger magnet spacing appears with the increase in *s* > 4 mm, which reduces the overall potential barrier height. Accordingly, it is essential to limit the reasonable working range of the adjustable distance *s* in structural design. In this study, the effective interval is determined as −2 mm to 4 mm.

**Figure 10:**
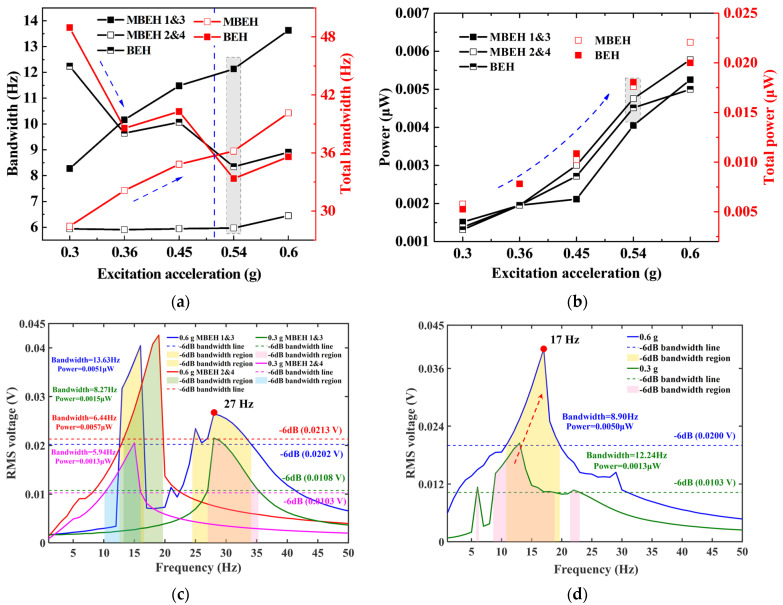
Performance comparisons under different excitation accelerations. (**a**) Subsystem bandwidth and total bandwidth of MBEH and BEH. (**b**) Subsystem power and total power of MBEH and BEH. (**c**) Voltage spectra of MBEH subsystems at 0.3 g and 0.6 g. (**d**) Voltage spectra of BEH subsystems at 0.3 g and 0.6 g.

After the above analysis, it can be concluded that MBEH achieves the optimal output performance at a magnet spacing of 15 mm, a beam length of 40 mm and an adjustable distance *s* of 3 mm. On this basis, the excitation acceleration is varied to compare the bandwidth and output power variation characteristics between MBEH and BEH, as presented in Figure 10. It can be seen from Figure 10a that the total bandwidth of BEH decreases gradually with the increase in excitation acceleration, while the total bandwidth of MBEH keeps rising and exceeds that of BEH at approximately 0.5 g.

As illustrated in Figure 10b, the output power of both structures increases with rising excitation. Under low-excitation conditions, BEH possesses a wider bandwidth, so the total output power of BEH is frequently higher than that of MBEH. Notably, when the excitation acceleration exceeds 0.54 g, the power growth rate of MBEH accelerates significantly, causing its overall power level to surpass that of BEH, and this performance advantage continuously expands with increasing excitation. This improvement demonstrates that the collaborative tuning mechanism enabled by the rhombus linkage mechanism is more advantageous under strong excitation, converting vibration energy into electrical energy more efficiently. During the low-to-medium-excitation stage, MBEH has not yet fully exerted its structural coupling effects, and the multi-peak response characteristics fail to manifest, resulting in a power performance inferior to that of BEH. In contrast, within high-excitation environments, the linkage tuning effect of the rhombus linkage mechanism is fully unlocked, and certain subsystem pairs form dual-peak vibrations. This behavior effectively counteracts the negative effects brought by stiffness hardening, thereby broadening the system operating frequency band and enhancing energy conversion efficiency to achieve the final performance inversion, which fully demonstrates the application advantages of MBEH structure under high-excitation vibration environments.

The voltage spectra of BEH and MBEH under 0.3 g and 0.6 g are analyzed in Figure 10c,d. The results reveal that BEH subsystems maintain single-peak vibration characteristics under all excitation conditions. Although increasing excitation drives a steady enhancement in output power, it also strengthens magnetic coupling and structural nonlinearity, which aggravates stiffness hardening. This effect steepens the frequency response curve of BEH, thereby narrowing its operating bandwidth. By contrast, by virtue of the rhombus linkage mechanism, MBEH coordinates magnet spacing and potential barrier height, effectively weakening the stiffness hardening effect and realizing continuous bandwidth expansion. Under 0.3 g excitation, all subsystems exhibit single-peak vibration patterns, resulting in relatively low overall system bandwidth and output power. When the excitation increases to 0.6 g, under the influence of mechanical coupling from the rhombus linkage mechanism, subsystem pairs 1,3 transition into a dual-peak response mode, which significantly broadens the effective operating frequency band and substantially increases power output. Meanwhile, constrained by the linkage deformation, subsystem pairs 2,4 still maintain narrow-band single-peak responses, yet they achieve relatively good power enhancement. This highly efficient regulation capability stems from the highly integrated design of the rhombus linkage mechanism. The four subsystems are linked via the mechanism, which enables the simultaneous optimization of potential barrier heights across all subsystems through a single tuning action. Consequently, as the excitation intensity varies, the coupled structure allows the subsystems to work synergistically across a wide excitation range without the need to design an independent potential barrier tuning mechanism for each individual subsystem.

**Figure 11:**
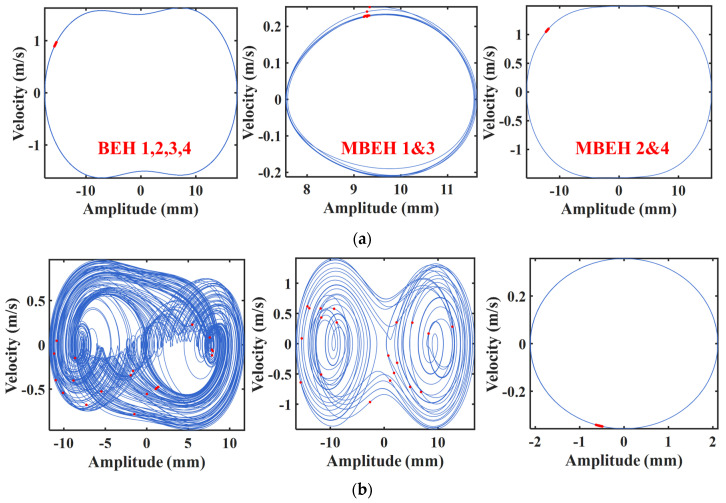

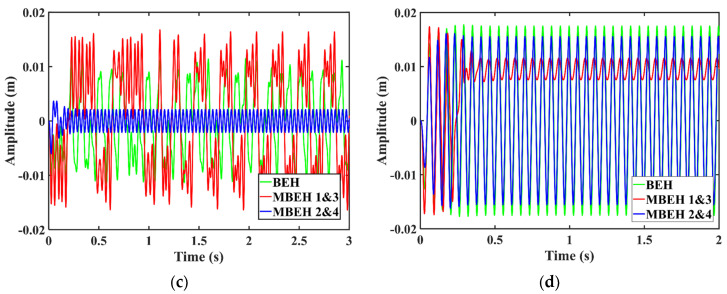
Phase trajectories, Poincaré sections (indicated by red dots), and time-domain responses of MBEH and BEH. (**a**) Phase trajectories and Poincaré sections at 17 Hz. (**b**) Phase trajectories and Poincaré sections at 27 Hz. (**c**) Time-domain responses at 17 Hz. (**d**) Time-domain responses at 27 Hz.

The phase trajectories, Poincaré sections, and time-domain responses of MBEH and BEH at 17 Hz and 27 Hz (Figure 10c,d) are compared and analyzed under an excitation acceleration of 0.6 g, with the corresponding results presented in Figure 11. At 17 Hz (the resonant frequency of BEH), as shown in Figure 11a,c, all BEH subsystems exhibit single-period large-amplitude inter-well vibrations. Their phase trajectories present perfect single closed loops, the Poincaré sections show single points, and the time-domain responses display maximum amplitudes with periodic regularity. Conversely, subsystem pairs 1,3 of MBEH exhibit period-doubling bifurcation, where the phase trajectories manifest as multiple nested closed orbits and the Poincaré sections display multiple discrete points. Although large-amplitude vibrations appear during the initial stage in the time domain, they attenuate rapidly and stabilize into periodic intra-well motions. However, subsystem pairs 2,4 still maintain single-period inter-well vibrations, characterized by smooth elliptical loops in the phase trajectories and single points in the Poincaré sections, with time-domain amplitudes slightly smaller than those of BEH subsystems but maintaining similar periodic regularity.

At 27 Hz (non-resonant frequency), as presented in Figure 11b,d, with the increasing frequency, BEH subsystems gradually enter chaos through a period-doubling bifurcation sequence, with the bifurcation critical frequency located between the two frequency points. This transition is characterized by a dispersed and folded phase trajectory, randomly distributed Poincaré points, and a limited time-domain amplitude. Conversely, MBEH exhibits response differentiation under the coupling of the rhombus linkage mechanism: subsystem pairs 2,4 exhibit period-1 intra-well motions with smooth closed phase trajectories, single Poincaré points, and lower amplitudes. Meanwhile, subsystem pairs 1,3 evolve into double-scroll chaotic attractors, featuring hierarchically nested phase trajectories, Poincaré sections with fractal structures, and amplitudes significantly higher than those of BEH subsystems. These variations indicate that the critical frequency for the degeneration bifurcation of subsystem pairs 2,4 from inter-well single-period to intra-well single-period motion lies between 17 and 27 Hz, whereas the critical frequency for subsystem pairs 1,3 to transition from period-doubling or intra-well states to large-amplitude inter-well chaos approaches 27 Hz. The difference in bifurcation critical frequencies between the two subsystem pairs reflects the complementary characteristics of potential barrier height and equivalent stiffness under the coupling effect of the rhombus linkage. This mechanism realizes energy redistribution among multiple subsystems, ensuring that the system still achieves an overall output performance superior to that of BEH subsystems even when deviating from the resonant frequency. This energy redistribution mechanism stems from the fact that the magnet spacings of the four subsystems are coupled by the same linkage mechanism, and their variations follow the cooperative relationship defined in Equation (8), thereby automatically coordinating the vibration states of each subsystem to effectively broaden the operating frequency band and improve energy harvesting efficiency.

## 4. Experimental Verification and Discussion

**Figure 12:**
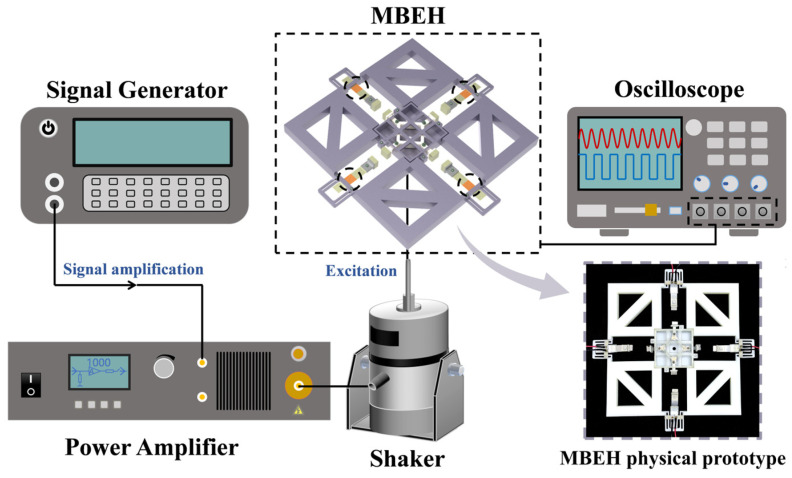
Experimental setup.

In order to verify the operational effectiveness of the proposed MBEH, an experimental test platform is established, as shown in Figure 12. The excitation signal is generated by a signal generator and amplified by a power amplifier to drive the vibration shaker for harmonic excitation. Under harmonic vibration excitation, MBEH converts mechanical vibration energy into electrical signals through piezoelectric patches. The main structural sizes and material parameters adopted in the experiment are consistent with the data listed in Table 1. To guarantee stable bistable dynamic responses of the system, a series of preliminary tests are carried out. Accordingly, the initial magnet spacing of the four subsystems is set to 17 mm, the cantilever beam length is fixed at 40 mm, and the adjustable distance is determined as *s* = 3 mm. On this basis, the influence of the rhombus linkage mechanism on the output performance of the energy harvester is further investigated.

It should be pointed out that the simulation selects excitation accelerations of 0.3 g and 0.6 g, whereas the experiment adopts 0.5 g and 1.9 g. This discrepancy primarily arises because the simulation represents an ideal state where the excitation acceleration acts directly on MBEH. In contrast, MBEH in the experiment must be fixed onto the vibration shaker via a fixture. The fixture itself possesses additional mass and introduces mechanical damping, which causes energy dissipation during vibration transmission. Consequently, higher nominal accelerations (0.5 g and 1.9 g) must be set in the experiment to overcome these non-ideal factors, ensuring that the effective excitation energy transferred to MBEH is sufficient to excite equivalent nonlinear dynamic behaviors observed in the simulations (0.3 g and 0.6 g). This study focuses on qualitatively revealing the potential barrier regulation mechanism of the rhombus linkage mechanism and the performance trends of MBEH relative to BEH subsystems, rather than quantitatively matching the absolute numerical values between simulations and experiments. Despite the different excitation levels, both simulations and experiments consistently demonstrate that MBEH achieves a broader operating bandwidth and higher output power than BEH subsystems. Thus, the comparative evaluation results remain highly reliable.

**Figure 13:**
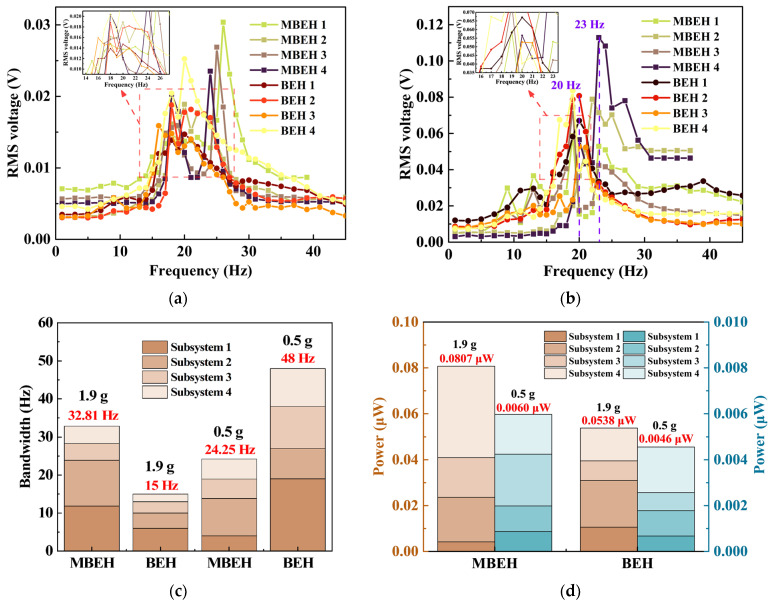
Voltage spectra, bandwidth, and power of MBEH and BEH under different excitation accelerations. (**a**) Voltage spectra of MBEH and BEH subsystems at 0.5 g. (**b**) Voltage spectra of MBEH and BEH subsystems at 1.9 g. (**c**) Subsystem and total bandwidth of MBEH and BEH under 0.5 g and 1.9 g excitation accelerations. (**d**) Subsystem and total power of MBEH and BEH under 0.5 g and 1.9 g excitation accelerations.

When the excitation accelerations are 0.5 g and 1.9 g, respectively, the voltage spectra, bandwidth, and power of MBEH and BEH are obtained, as shown in Figure 13. Due to installation errors in each beam, the output waveforms are not exactly the same. The resonant frequencies of BEH subsystems remain unchanged, while the resonant frequencies of MBEH shift, as depicted in Figure 13a,b. This frequency shift occurs because the rhombus linkage mechanism couples the magnet spacings of the four subsystems together. As the excitation increases, the geometric deformation of the rhombus linkage mechanism causes the subsystem spacings to vary synchronously according to the law defined in Equation (8), thereby altering the equivalent stiffness and potential barrier height of the system. Consequently, the resonance peak positions of MBEH shift with the excitation intensity, whereas BEH subsystems maintain a fixed magnet spacing and an unchanged potential barrier, exhibiting characteristics approximation of a linear system.

A detailed analysis of the bandwidth and power of MBEH and BEH reveals that at an acceleration of 1.9 g, the total bandwidth of MBEH is 32.81 Hz, which is 2.19 times that of BEH subsystems, as depicted in Figure 13c. At an acceleration of 0.5 g, the smaller excitation results in a lower total bandwidth for MBEH compared to BEH subsystems, which is consistent with the simulation analysis in Figure 10a. Correspondingly, at an excitation acceleration of 1.9 g, the output power of MBEH is 0.0807 μW, which is 1.5 times that of BEH subsystems, as depicted in Figure 13d. At an excitation acceleration of 0.5 g, the output power of BEH subsystems is approximately 23.33% lower than that of MBEH. Experimental data indicate that the potential barriers of each subsystem in MBEH are tuned to appropriate heights, and the excitation energy is sufficient to drive the system into frequent inter-well transitions or chaotic motions. These nonlinear dynamic behaviors enrich the frequency components of the response, thereby significantly broadening the effective operating frequency band. The reason why the bandwidth of MBEH is inferior to that of BEH subsystems under weak excitation is that the excitation energy falls below the potential barrier threshold, trapping the system within a single potential well to perform approximate linear vibrations, which prevents the manifestation of its nonlinear advantages.

**Figure 14:**
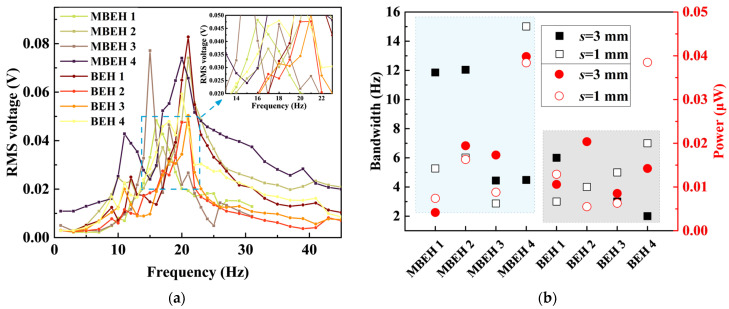
Voltage spectra, bandwidth, and power of MBEH and BEH under 1.9 g excitation acceleration when adjustable distance *s* = 1 mm. (**a**) Voltage spectra of MBEH and BEH subsystems. (**b**) Comparison of bandwidth and power for MBEH and BEH under *s* = 1 mm and *s* = 3 mm.

At an excitation acceleration of 1.9 g, the voltage spectra, bandwidth, and power of MBEH subsystems and BEH subsystems at *s* = 1 mm are analyzed and compared with the results at *s* = 3 mm, as shown in Figure 14. Figure 14a shows that when *s* = 1 mm, the resonant frequency offset of MBEH is small (with a maximum offset of only 4 Hz), while its output performance remains significantly superior to that of BEH subsystems. This small frequency shift occurs because the stiffness change of the structure is limited at a small adjustable distance, which weakens the effect on the resonant frequency. However, even a minor change in spacing is sufficient to generate differences in the potential barrier heights of each subsystem, driving them into distinct nonlinear dynamic states. For instance, some subsystems remain in intra-well periodic motions, while others begin to exhibit inter-well transitions or period-doubling motions. This differentiated nonlinear response ensures that the total output power of MBEH remains higher than that of BEH subsystems.

It is observed from Figure 14b that the bandwidth and power fluctuation ranges of BEH subsystems are narrow. In contrast, benefiting from the linkage effect of the rhombus linkage mechanism, the differences in bandwidth and output power among MBEH subsystems are more pronounced. This phenomenon is a direct result of the interrelated magnet spacings among the four subsystems, meaning their dynamic responses are no longer independent, and the energy is redistributed according to the geometric relationships of the rhombus structure. Consequently, although the performance of individual subsystems fluctuates, the overall system output is significantly optimized. By comparing the two working conditions of *s* = 1 mm and *s* = 3 mm, it can be found that the power distribution law among MBEH subsystems remains highly consistent, and the fluctuation amplitude does not change significantly. This consistency indicates that within a small adjustable distance range, the regulation mode of the rhombus structure on the subsystem output power possesses excellent stability. Regarding the bandwidth, the differences among MBEH subsystems under both conditions are also much larger than those of BEH subsystems, and the overall bandwidth level remains significantly higher. This trend demonstrates that even at a small adjustable distance of *s* = 1 mm, the rhombus mechanism can still effectively drive differentiated responses among subsystems to achieve operating frequency band broadening. Compared with the simulation results, the experimental tests verify that MBEH still exhibits superior output performance even at a small adjustable distance, which further confirms the structural advantages of the mechanism in working condition adaptability.

**Figure 15:**
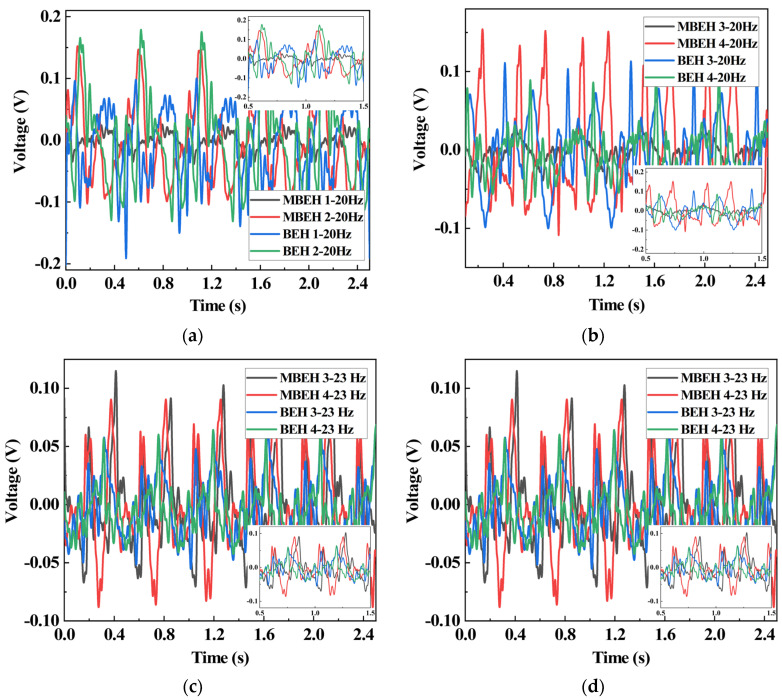
Time-domain responses of MBEH and BEH at 20 Hz and 23 Hz. (**a**) Subsystems 1 and 2 of MBEH and BEH at 20 Hz. (**b**) Subsystems 3 and 4 of MBEH and BEH at 20 Hz. (**c**) Subsystems 1 and 2 of MBEH and BEH at 23 Hz. (**d**) Subsystems 3 and 4 of MBEH and BEH at 23 Hz.

The time-domain responses of MBEH and BEH at the frequency points of 20 Hz and 23 Hz in Figure 13b are shown in Figure 15a–d. At 20 Hz (close to the resonant frequency of BEH subsystems), the output amplitudes of subsystem 2 and subsystem 3 of BEH are approximately 0.18 V and 0.1 V, respectively. Meanwhile, the output amplitudes of subsystem 2 and subsystem 4 of MBEH reach 0.152 V, with their waveforms presenting sharp pulses that indicate a large-amplitude vibration state. Conversely, the output amplitudes of subsystem 1 and subsystem 3 of MBEH are low, with flat waveforms indicating that they are trapped in an intra-well micro-vibration state. These results are consistent with the simulation analysis in Figure 11c,d, reflecting the fluctuating output distribution characteristics of different MBEH subsystems.

A frequency of 23 Hz is close to the resonant frequency of MBEH subsystems, at which the output of each MBEH subsystem exceeds that of BEH subsystems. Specifically, subsystem 1 achieves the highest output of approximately 0.112 V, and the amplitudes of subsystems 2 and 4 are also prominent (approximately ±0.1 to 0.11 V). Their waveforms exhibit distinct distortions and multi-peak superpositions, indicating that the system undergoes complex motions with superimposed frequency components, which is a common phenomenon in nonlinear systems under relatively strong excitations. In contrast, the output of each BEH subsystem remains low (only ±0.05 to 0.07 V), and the corresponding waveforms are relatively regular.

The consistent variation trends between the simulations and experiments firmly indicate that the rhombus linkage correlates the magnet spacings of the four subsystems through geometric linkage according to Equation (8), which leads to differences in the potential barrier heights and equivalent stiffness of each subsystem. Consequently, under different frequencies, distinct subsystems automatically assume the primary vibration output, thereby achieving energy transfer among the subsystems and maintaining the overall output at a high level across a wide frequency range. This high output directly stems from the multi-subsystem collaborative response enabled by the highly integrated design. The consistent superiority at different frequencies firmly demonstrates the exceptional output performance advantages of the mechanism under varied working conditions.

**Figure 16:**
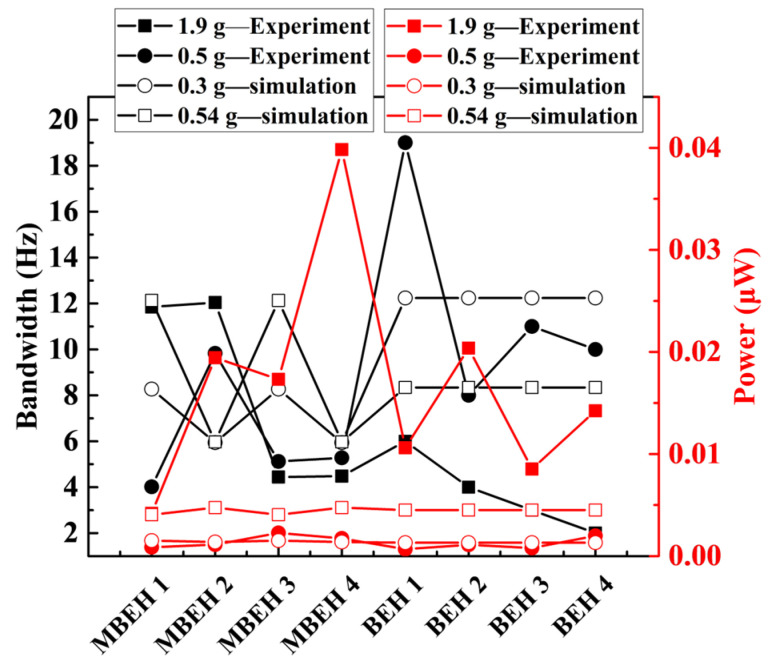
Bandwidth and power of MBEH and BEH subsystems under simulation (0.3 g, 0.54 g) and experimental (0.5 g, 1.9 g) excitation conditions.

Figure 16 compares the bandwidth and power of MBEH subsystems and BEH subsystems under simulation (0.3 g, 0.54 g) and experimental (0.5 g, 1.9 g) excitation. The results show that the output variation trends of each MBEH subsystem in simulation and experiment remain highly consistent. Subsystem pairs 1,3 and subsystem pairs 2,4 present symmetrical distribution characteristics respectively, and the variation trends of the two groups show an inverse correlation. The overall output amplitude of experimental results is higher, which mainly stems from the larger excitation acceleration adopted in the experiment. Both simulations and experiments confirm that MBEH enables energy redistribution among subsystems through the collaborative coupling of the rhombus linkage, ensuring that the total bandwidth and total power are superior to those of traditional BEH subsystems across a wide frequency range. This consistency validates the reliability of the performance advantages of the rhombus linkage mechanism under different excitation conditions.

Furthermore, beyond the macro-performance trends of bandwidth and power, the resonant frequency characteristics also deserve a detailed discussion, taking the fundamental natural frequency of approximately 17 Hz obtained from COMSOL Multiphysics 6.1 eigenfrequency study as a baseline. In the aforementioned MATLAB Simulink (R2018b) nonlinear dynamic simulations, the resonant frequency of BEH remains essentially unchanged around 17 Hz baseline. In contrast, during MBEH simulation, the resonant frequency of each subsystem shifts significantly from this baseline. This deviation is driven by the coupling effect of the rhombus linkage mechanism and the nonlinear magnetic repulsive force. A similar frequency shift phenomenon is observed experimentally. Although the resonant frequency of BEH varies slightly due to installation errors, the overall deviation remains minimal. Conversely, the resonant frequencies of MBEH subsystems exhibit more pronounced shifts relative to the simulation values, and the frequency scatter among different subsystems becomes more conspicuous.

The discrepancies in frequency shift trends between simulations and experiments stem from the ideal symmetry assumptions in the numerical model, such as rigid rods and the magnetic dipole approximation. In the experiments, however, unavoidable uncertainties like installation misalignments and assembly tolerances break this symmetry, causing the measured resonant frequencies to deviate from the simulated predictions. Nevertheless, both numerical and experimental results consistently confirm that MBEH delivers a wider operating bandwidth and higher output power than BEH. Crucially, the primary objective of this study is to qualitatively reveal the potential barrier regulation mechanism of the rhombus linkage and demonstrate the performance trends, rather than pursuing precise quantitative frequency matching.

To bridge the gap between numerical simulations and experimental results, model updating and parameter identification methods will be implemented in future work. Specifically, the simulation model can be calibrated by back-calculating key parameters using the experimentally measured resonant frequencies of each subsystem as the target metrics. These key parameters encompass the equivalent elastic modulus of each cantilever beam, the equivalent coupling stiffness of the rhombus linkage mechanism, and the coefficients of the magnetic force model. After identifying the dominant parameters via sensitivity analysis, optimization techniques such as least-squares estimation or genetic algorithms will be utilized to solve for the optimal parameter combination that matches the experimental dataset. Consequently, this model updating process will precisely align the simulation with the measured resonant frequency trends, thereby significantly enhancing the predictive fidelity of the numerical framework for broadband performance evaluation.

**Figure 17:**
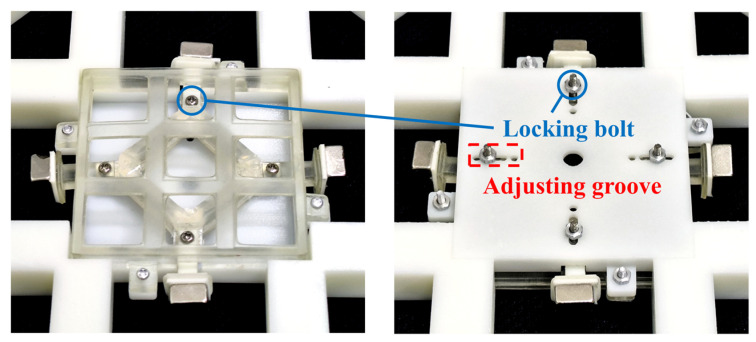
Components of displacement regulation mechanism for MBEH.

Additionally, the detailed adjustment operations of the rhombus linkage mechanism were recorded during the experiment. As illustrated in Figure 17, the displacement regulation mechanism of MBEH integrates the four bistable subsystems into a single unit via the rhombus linkage mechanism. The mechanism is positioned on a support platform, and the fixture features built-in adjusting grooves. The four connecting corners of the rhombus linkage mechanism are fixed through locking bolts to achieve precise displacement control. Due to the rigid linkage characteristic of the mechanism, adjacent vertices undergo opposite displacements while diagonal vertices move in the same direction. Consequently, the operator only needs to loosen two bolts, move them to the target positions, and lock them again. This simple operation simultaneously changes the magnet spacings of the remaining three subsystems through linkage transmission, and the entire adjustment process takes only approximately 10 s. This integrated design significantly reduces the complexity of potential barrier regulation, while effectively enhancing the structural integration and dynamic adaptability of the system. For comparison, employing the conventional independent tuning mechanism to simulate four BEH subsystems requires separately adjusting the grooves of each corresponding BEH subsystem and repeatedly observing each channel output via an oscilloscope to guarantee consistent bandwidth coverage. This conventional process takes more than 3 min in total. This direct contrast clearly validates the prominent advantages of the rhombus linkage mechanism in terms of tuning convenience and high integration.

Meanwhile, the normalized power density (*NPD*) is adopted to evaluate the performance of MBEH. This standardized indicator can be utilized to compare the performance of energy harvesters under different dimensions and excitation conditions. The NPD is defined as follows [54]:

(13) $$NPD=\frac{p_{rms}}{V\cdot a_{rms}^{2}}$$

where *P_rms_* is the root-mean-square (RMS) value of the output power (which refers to the total power in this study), *V* represents the total volume of the harvester, and *a_rms_* denotes the RMS value of the excitation acceleration. The unit of *NPD* is W∙s^4^∙m^−5^ or mW∙cm^−3^∙g^−2^.

Under an excitation of 1.9 g (approximately 18.62 m/s^2^), the *NPD* of MBEH is approximately 4.15 × 10^−5^ W∙s^4^∙m^−5^ (approximately 0.00415 mW∙cm^−3^∙g^−2^), whereas the corresponding value for BEH subsystems is 2.76 × 10^−5^ W∙s^4^∙m^−5^ (approximately 0.00276 mW∙cm^−3^∙g^−2^). The *NPD* of MBEH is approximately 1.5 times that of BEH subsystems, demonstrating that under the same volume and excitation conditions, the rhombus linkage can significantly enhance the energy conversion capability per unit volume.

It should be pointed out that flexible PVDF piezoelectric films are adopted in this study, whose piezoelectric constant *d*_31_ is lower than that of PZT piezoelectric ceramics. PVDF possesses a high intrinsic resistance and interface contact resistance, which generates continuous charge dissipation during dynamic vibration processes. Furthermore, the cantilever substrate is made of 304 stainless steel with high stiffness, resulting in limited bending strain under a 1.9 g excitation. Considering the aforementioned factors, the experimental output voltage and power in this study remain within the ranges of tens to hundreds of millivolts and microwatts, respectively, which represents a normal performance for the combination of PVDF and stainless steel beams under low-frequency, low-excitation conditions. Although the absolute numerical values are relatively small, both MBEH and BEH subsystems are measured under completely identical conditions, and their relative performance remains prominent, thereby fully validating the practical advantages of the rhombus linkage mechanism.

## 5. Conclusions

This paper proposes a novel multi-subsystem bistable vibration energy harvester (MBEH) that adopts a rhombus linkage mechanism to enable tunable potential barrier height. The rhombus linkage mechanism realizes the coordinated motion of four bistable subsystems to adjust the potential barrier height of the overall system, which effectively solves the limitation of traditional fixed-barrier bistable structures that struggle to adapt to broadband and variable excitation environments. The core innovation of this paper can be summarized as the proposal of a synchronous potential barrier regulation mechanism for multiple subsystems driven by the rhombus linkage mechanism. Consequently, this mechanism yields significant performance advantages, achieving highly efficient coupling among subsystems. Furthermore, potential barrier regulation efficiency is substantially increased, while operating bandwidth of subsystems is superimposed. The lumped parameter theoretical model and magneto-electro-mechanical coupling dynamic equations are established, and the geometric linkage characteristics as well as the potential barrier adjustment principle of the rhombus linkage mechanism are demonstrated. Numerical simulations and experimental tests are conducted to analyze the effects of magnet spacing, cantilever beam length, excitation acceleration, and adjustable distances on the system bandwidth, output power, and dynamic responses.

The simulation results show that the rhombus linkage structure significantly improves the potential barrier adjustment efficiency. Under an excitation of 0.6 g, the summed subsystem bandwidth of MBEH is increased by 13.58 Hz, and the output power rises by 0.0223 μW compared with conventional BEH. The experimental results indicate that the summed subsystem bandwidth of MBEH reaches 32.81 Hz, which is 2.19 times that of BEH, and its output power is approximately 1.5 times that of BEH under a strong excitation of 1.9 g.

Both experimental and numerical results consistently verify that the designed MBEH possesses superior broadband adaptability and energy harvesting capacity under both weak and strong excitations. This study provides a novel, highly integrated, and high-efficiency solution for self-powered supply of distributed IoT sensors.

Meanwhile, MBEH still faces several challenges in practical applications: its relatively large volume and mass restrict miniaturization, and the manual tuning mechanism requires upgrading to an adaptive self-regulation system. Furthermore, assembly tolerances of each subsystem demand higher batch consistency, and the absolute output power remains small due to the low piezoelectric constant of PVDF and high cantilever substrate stiffness. Additionally, the presented model is mainly intended to capture the underlying mechanism and performance trends of the multi-subsystem coupling, rather than to provide an exact quantitative prediction of the experimentally observed resonant frequencies. Future work will focus on structural miniaturization, adaptive self-tuning, and optimizing beam structures and substrate materials in combination with high-performance piezoelectric materials to maximize strain and power output.

## Figures and Tables

**Table 1:** Key structural parameters of MBEH.

Parameter		Value
Cantilever (stainless steel)	Elastic modulus *E_C_* (GPa)	194
	Density *ρ_C_* (kg/m^3^)	7930
	Length *L_i_* * (m)	0.040
	Width W (m)	0.010
	Thickness H (m)	0.0002
Magnets (NdFeB)	Residual flux density *B_r_* (T)	1.45
	Density *ρ_m_* (kg/m^3^)	7500
	Length *L_m_* (m)	0.0092
	Width *W_m_* (m)	0.0092
	Thickness *H_m_*	0.0054
	Elastic modulus *E_m_* (GPa)	160
	Magnetization intensity *M_A_*, *M_B_* (10^6^ A/m)	11.539
	Magnet volume *V_A_*, *V_B_* (10^−7^ m^3^)	4.5706
Piezoelectric material (PVDF)	Elastic modulus *E_P_* (GPa)	2.5
	Density *ρ_p_* (kg/m^3^)	1780
	Length *L_P_* (m)	0.010
	Width *W_P_* (m)	0.010
	Thickness *H_P_*	0.00003
	Piezoelectric constant *d*_31_ (−10^−12^ C/N)	17
	Dielectric constant *ε*_33_ (10^−11^ F/m)	8.4
	Elastic compliance coefficient $s_{11}^{E}$ (10^−10^ m^2^/N)	4
	Intrinsic capacitance *C_p__i_* * (10^−10^ F)	4
Subsystem	Equivalent damping *C_eqi_* * (N·s/m)	0.1
	Equivalent resistances *R_i_* * (10^5^ Ω)	3.2
	Gravitational acceleration *g* (m/s^2^)	9.8

* *i* = 1, 2, 3, 4 denotes the subsystem number.

## Data Availability

The data presented in this study are available on request from the corresponding author.
